# Longitudinal monitoring of workloads in women's division I (DI) collegiate basketball across four training periods

**DOI:** 10.3389/fspor.2023.1108965

**Published:** 2023-04-11

**Authors:** Randy Towner, Abigail Larson, Yong Gao, Lynda B. Ransdell

**Affiliations:** ^1^ Women’s Basketball Program, University of Utah, Salt Lake City, UT, United States; ^2^ Department of Kinesiology and Outdoor Recreation, Southern Utah University, Cedar City, UT, United States; ^3^ Department of Kinesiology, Boise State University, Boise, ID, United States

**Keywords:** catapult, sport technology, female athlete, overtraining, periodization, sport performance

## Abstract

Women's collegiate basketball is a fast-growing, dynamic sport that spans 8 or more months, with athletes competing in 30 + games in a season. The aim of this study was to quantify and profile the external load of practices and games during a Power-5 DI Women's Collegiate Basketball season. Specifically, Average PlayerLoad (PL), PlayerLoad per minute (PL*min^−1^), High Inertial Movement Analysis (High-IMA), and Jumps were quantified using Catapult Openfield software during four distinct training periods of the year: 8-hour preseason, 20-hour preseason, non-conference, and conference game play. Weekly variations and acute to chronic workload ratios (ACWR) were also examined. Eleven subjects participated in daily external load monitoring during practice and games *via* Catapult's ClearSky T6 inertial measurement units (IMU). Averages, standard deviations, and confidence intervals were calculated for training period comparisons, and Cohen's *d* was calculated as a measure of effect size. Findings include normative values to provide context for the demands experienced across an entire season. PL was significantly higher during non-conference play than during any of the other three training periods (*p* < 0.05). Descriptive data enumerate percent change and ACRW variations throughout the season. These data can be used to describe the physical demands across a season and provide physical profile guidelines for coaches.

## Introduction

Women's collegiate basketball has increased in popularity, with a concurrent increase in player fitness demands and game performance expectations. The DI Women's Basketball season in the National Collegiate Athletic Association (NCAA) spans from August to March with 30 + regular season games. Teams can begin mandatory on-campus workouts in August, and official pre-season practices in late September, with game play beginning in November. To ensure athlete safety and promote academic success, the NCAA has guidelines regulating practice duration and athlete requirements. NCAA regulations are sport season-dependent, and these include offseason, preseason (which can be broken into 8-hour preseason and 20-hour preseason), non-conference game play, and conference game play. The offseason includes summer, while preseason includes early fall (where no official games are played), also referred to as the 8-hour period and the 20-hour period. In the 8-hour period, NCAA guidelines limit required training to eight hours of activity per week; four of those hours may be used on the court for basketball activity, while the other four can be used for other activities such as strength and conditioning. Once teams have been approved to start official practices, usually about a month before the first game, they move into a 20 h per week pre-season period. During this time, coaches can use those 20 h at their discretion, but athletes are required to take two days off per week. Once teams begin playing games, only one day off per week is required. This portion of the season is termed non-conference game play, or the non-conference schedule, because teams play outside of their designated conference and include any DI schools that agree to play each other. During this portion of the season, games can be played any day of the week and travel schedules are highly variable. During conference play, teams compete on the same two days of the week, creating a more consistent schedule; one day off per week is still required. Often athletes are participating in mandatory workouts for at least 7 months of the year, and in many cases, more. Training blocks of this length are challenging to periodize and many coaches struggle to appropriately stimulate player fitness without increasing risk of burnout or overuse injuries. Issues such as sudden spikes in workload and lack of training variation are of particular concern to the long-term health and performance of the athletes (2, 3, 10, 11, 34).

Workload monitoring allows coaches to quantify the demands placed on athletes and make evidence-based decisions in an effort to reduce risk of injury and burnout and optimize training and peaking. With the desire to quantify training and competition demands, coaches and trainers utilize an array of variables to estimate workload and inform short- and long-term training progressions. Intrinsic (internal) variables include psychological constructs such as rating of perceived exertion (RPE) (4, 9, 35), and physiological constructs such as heart rate (HR), and blood lactate (15). Extrinsic (external) variables include time motion analysis (TMA), and global positioning systems (GPS) (10, 11, 34). More recently, inertial measurement units (IMU) have been used to provide a multidimensional view of training-related physical demands, and they are considered the “gold standard” for reliable and valid measures of athlete workload (15). An IMU brings together triaxial accelerometers (assesses movement in three dimensions), gyroscopes (determines orientation), and magnetometers (measures directions) (10, 29) into one small wearable device to measure external loads (Catapult Clearsky T6, Melbourne, Australia). This allows tracking of movements such as accelerations, decelerations, changes of direction, jumps, and directionality of movements (39). IMU's measure workload data as arbitrary units (AU); AUs combine all movements into a single number that represents the intensity and duration of the workload session; in Catapult, this measurement is termed PlayerLoad (PL). Other variables typically examined in studies profiling women's basketball include PlayerLoad per minute (PL*min^−1^), a measure of intensity over time; High Inertial Movement Analysis (High-IMA), a measure of fast (above 3.5 m*s^−1^) accelerations, decelerations, changes of direction, and free running; and Jumps (29). Expanded definitions of the variables used in this study, and by others conducting workload analyses in women's basketball, are summarized in Table 1.

**Table 1:** Common workload variables.

Variable	Calculated Method
8-hour preseason	Offseason period where athletes can practice 4 h on the court & 4 h for S&C, film, ECT
20-hour preseason	20-hour practice period that begins one month before games start
Non-Conference	Practice and games against non-conference opponents
Conference	Practice and games against conference opponents
Arbitrary Units (AU)	Arbitrary Units (AU) are used as a scaling factor to compare the total volume of work performed. Specifically, they are the units produced by Catapult® devices to calculate PL, PL.min^−1^, High-IMA, and other metrics available.
PlayerLoad (PL)	PlayerLoad is an overall indicator of work performed during activity that includes movements in all planes. PL is defined as “a modified vector magnitude, expressed as the square root of the sum of the squared instantaneous rate of change in acceleration in each of the three vectors (*x*, *y* and *z* axis)—and divided by 100; it is represented in arbitrary units (AU).” (7). The formula for PlayerLoad is listed below “where *ax_i_*, *ay_i_* and *az_i_* are the acceleration values in *x*, *y* and *z* directions respectively, and *i* = 0…, *n* represents the sampled accelerometer points with *n* + 1 points over the time of the session” (see https://support.catapultsports.com/hc/en-us/articles/360000574716-What-is-Player-Load-) for more information. $\frac{\sum_{i=1}^{n}\sqrt{{\left(ax_{i}-ax_{i-1}\right)}^{2}+{\left(ay_{i}-ay_{i-1}\right)}^{2}+{\left(az_{i}-az_{i-1}\right)}^{2}}}{100}$
PlayerLoad Per Minute (PL*min^−1^)	PlayerLoad Per Minute is PL divided by minutes of activity. PL*min^−1^ is a measure of intensity.
High Inertial Movement Analysis (High-IMA)	High-IMA is a metric that combines accelerations, decelerations, change of direction, and free running when acceleration is >3.5m·s^−1^.
Jumps	Total number of jumps
Total Weekly Training Load	Sum of weekly PL for training and games
Weekly Training Load (acute)	Average workloads attained over the previous 7 days
Chronic Workload	Rolling average of training load experienced in the previous 4 weeks
Acute: Chronic Workload Ratio (ACWR)	Acute (1 week) workload divided by chronic (4 week) workload

Player Load, PL.min^−1^, High-IMA, Weekly Training Load, Chronic Workload, and Acute: Chronic Workload Ratio are in arbitrary units (AUs). Jumps are the raw number of jumps performed. These are the standard metrics for reporting Catapult workload data.

The Catapult system used in this study adds a Local Position Measurement (LPM) system to enhance information provided by IMU's. LPMs are particularly helpful when determining the demands of indoor training or competition sessions, such as those conducted on a basketball court. Data from the Catapult system quantify fluctuations in volume through AU workloads across days, weeks, and the course of the season. Acute fluctuations in workload, represented by percent change, are one way to quantify load. Acute to chronic workload ratio (ACWR, or acute load–the sum of the last seven days, divided by chronic load–the sum of the previous four weeks), is another strategy for quantifying load. From a theoretical perspective, acute workload indicates fatigue and chronic workload indicates fitness (21). While percent change and ACWR are not without criticism (14, 20, 21) these metrics, along with other IMUs, are typically part of an overall performance monitoring and improvement plan that may consider multiple factors (e.g., sleep, nutrition, stress). Monitoring ACWR as part of a multifactorial plan facilitates athlete development across their training age or years of participation (13), informs workload adjustments to minimize fatigue and burnout (36, 37), and it has been previously associated with a lower risk of non-contact injuries (3). Specifically, prior research has established that when RPE's and AU's show workload increases of ≥15% compared to the previous week, injury risk can increase between 28% and 49% (13). In addition, ACWR between 1 and 1.5 is associated with the lowest injury rate (36%) when compared to other ranges (<0.5; 54%), (0.5–.99; 51%), and (>1.5; 59%) (33, 36). A recent systematic review by Andrade and colleagues (3) concluded that 90% of the studies reviewed suggest that athletes are at higher injury risk when ACWR is higher–vs. lower or moderate. Quantification of these variables provides coaches with additional information for evidence-guided training, competition, and recovery decisions.

IMU data from athletes participating in a variety of sports have been examined. Specifically, related research during basketball play has included workload monitoring methods (10, 11, 23, 34), workloads in games vs. practices (12), workload requirements between various playing positions and drills (1, 23, 31, 32), the effect of fluctuating training loads on performance variables (4, 16, 17) and pre-season (16) and in-season workload demands (9). However, the previously mentioned studies examined short-term workloads. Only a handful of studies have examined season-long or longer-term workload variables. Game data over the course of four years (29), in-season weekly load fluctuations utilizing RPE (25), and neuromuscular response to training loads (26) have been examined among women's basketball players. In addition, in-season weekly load fluctuations utilizing RPE (9) and 10 weeks of pre and in-season play utilizing HR (4) have been examined; however, these studies were conducted with male basketball players. It is unknown how women's basketball loads, measured utilizing IMU data, vary over the course of the entire season, and in particular how demands may fluctuate between pre-season and in-season play. There is a need for long-term studies quantifying changes in workload among women's basketball players (11, 29) in order to optimize training programs to meet the demands of the competition season.

While value can be drawn from existing research, variables used to quantify basketball workload and workload monitoring vary (25, 26, 29), and only one study has examined IMU's among women basketball players over the course of a whole year (26). Interestingly, the primary purpose of the study by Peterson and Quiggle (26), was to examine the neuromuscular response to training loads-not to examine training load variations across a year. Petway et al. (27) have called for the need to examine how different workload metrics vary throughout a season. Therefore, given the lack of longitudinal research on seasonal workload variations in women's basketball, the purpose of the present study was to quantify the external load of practices and games during a Power-5 DI Women's Collegiate Basketball season, while specifically examining the fluctuations and differences in loads between and within four key periods: 8-hour preseason, 20-hour preseason, Non-Conference, and Conference play. IMU data (Catapult Innovations, Melbourne, Australia) were utilized to determine weekly team averages of PL, PL^.^minute^−1^, High-IMA, and Jumps as well as weekly percent change, and ACWR. Data collected for this study provides information for physical profiling of this important group of athletes.

## Materials and methods

### Experimental design

A longitudinal, retrospective, and observational study design was used to examine four IMU metrics across four distinct periods: 8-hour preseason, 20-hour preseason, Non-Conference play, and Conference play. Data captured *via* Catapult T6 wearable devices were regularly collected by the sports performance coaches at a large urban university in the western USA. Data were available from eleven female basketball athletes over the course of the 2019–2020 season.

The variables used for analysis in this study were included in previous research on women's Division I basketball players (29), and recommended in a recent systematic review on external workload monitoring in team sports (15). PlayerLoad (PL), an indicator of overall work, PlayerLoad per minute (PL*min^−1^), an indicator of the intensity of effort over time, total high inertial movement analysis (High-IMA), a metric that combines accelerations, decelerations, change of direction, and free running when acceleration is greater than or equal to 3.50 m*s^−1^*s^−2^, and total jumps (Jumps), were used as the primary measures for this study. PL was further examined to calculate weekly training loads (i.e., weekly percent difference) and an acute (7 days) to chronic (28 days) workload ratio (ACWR). These variables are described in detail in Table 1. All variables were measured using Catapult ClearSky T6 (Catapult Innovations, Melbourne, Australia), and analyzed using Catapult OpenField Software.

### Subjects

Participants included eleven (*n* = 11) DI female basketball players at a Power-5 DI University located in the West. Athletes ranged from 18 to 24 years (mean = 20.2 ± 1.3 years), and participated in 102 practices, and 30 games. They were tracked during all mandatory basketball activities, and variables were de-identified. Data were previously collected as part of the University's Basketball team daily monitoring practices. Approval from the Institutional Review Board (IRB #08-062020a) was obtained and each athlete gave written consent to use their previously collected and de-identified data. All participation was voluntary.

### Procedures

#### Match play demands

Practice and game sessions were held in University gyms. In order to quantify the physical demands throughout the season, the inertial measurement unit (IMU) was utilized and placed on each athlete. Each athlete wore a sports vest which housed a small pouch where the unit was placed, located at the top of her back between the scapulae. As the athlete entered the gym each day, she would receive the same IMU device assigned at the beginning of the year. The first author would then assist to place the device in the vest and ensure the device was turned on and tracking movements. While in practice, the investigator would live track the practice or game utilizing Catapult software (Openfield; Catapult Innovations, Melbourne, Australia) to indicate when drills began and ended. Non-activity time that exceeded two minutes, including teaching, timeouts, and water breaks, was excluded from the study. Athletes who did not participate in the full practice, were injured that day, or did not play more than 10 min in the game were also excluded from that day's data. Data were downloaded utilizing Openfield software and exported to Microsoft Excel (Redmond, WA). Catapult wearable devices have demonstrated acceptable reliability and validity in soccer (8), indoors (22), and during treadmill running (5).

Researchers have examined intra- and inter- device reliability in activities that closely resemble basketball (24). When examining intra-device reliability (a) the majority of CV (coefficient of variation) values for various directions and levels of acceleration were low (e.g., < 1.0%, although the values ranged from 0.01% to < 3.0%), and (b) ICCs ranged from 0.77 to 1.0 (very large to nearly perfect) (22). Taken together, CVs and ICCs indicate a high level of intra-device reliability. Inter-device differences were noted for various directions and levels of acceleration, such that effect sizes ranged from 0.54 (medium) to 1.20 (large), indicating a lower level of inter-device reliability compared to intra-device reliability (24).

Criterion Validity of the Catapult ClearSky T6 local positioning system (LPS) was examined by asking participants to complete a circuit of 7 movements that simulated team sport movements, and comparing results to a Vicon motion capture system (considered the gold standard) (18). The root mean square error was 0.20 ± 0.05 m and mean bias detected an overestimation for all distance bands. While there is some error, the LPS system demonstrates acceptable accuracy for measuring inter-unit distance. Concurrent validity was tested by Nicolella et al. (24), who concluded that the difference between accelerometer measured peak acceleration and the Catapult device ranged from 1.0% to 23.5%. A consistent difference of 15% was reported, with PlayerLoad from Cataput being consistently lower than load calculated using a Cartesian formula. While there have been questions about reliability and validity of these devices, there is agreement that inter-unit reliability is high (24), and that Catapult devices are useful tools for assessing athlete workload.

### Statistical analysis

To establish normative values for PL, PL*min^−1^, High-IMA, and Jumps over the course of the four time-periods (8-hour preseason, 20-hour preseason, Non-Conference, and Conference), means and SDs were calculated. To investigate whether there were any differences in the performance of PL, PL*min^−1^, High-IMA, and Jumps across the four periods (i.e., 8-hour preseason, 20-hour preseason, Non-Conference, and Conference) of the training season, a series of linear mixed model analyses were conducted. This approach was necessary to account for the nested structure of the data, where repeated observations were obtained from the same players across the four periods, and to control for potential statistical errors. Using linear mixed models, it was possible to estimate the effects of predictor variables and determine whether there were significant differences in performance across the different periods of the training season. Using one of the linear mixed model analyses in this study as an example, Jumps were the dependent variable, and the periods of the training season were the predictor variables. By accounting for the correlation between repeated observations of Jumps on the same players across the four training periods, the model was able to control for statistical errors. Therefore, the use of linear mixed models in this study allowed for a more rigorous analysis of the data, enabling the identification of any significant differences in the performance of PL, PL*min^−1^, High-IMA, and Jumps across the different periods of the training season. Bonferroni *post hoc* analyses were further conducted to determine specific significant differences among the comparisons of the four periods. Significance level was set at 0.05. Cohen's *d* was also utilized to determine effect sizes, with 0.2–0.49 categorized as small, 0.5–0.79 as medium, and 0.8 and above as large. Confidence intervals were set at 95%.

To establish season-long weekly descriptive values for weekly PL totals, weekly percent change in PL, and ACWR, data were calculated and graphed for visual inspection. Percent change was calculated [(new week − previous week)/previous week]) and graphed along with weekly PL totals. In this study, ACWR was calculated utilizing an exponentially weighted equation (29). $\mathrm{EWM}A_{\mathrm{today}}=\;\mathrm{Loa}d_{\mathrm{today}}*\;2/(N+1)+((1-(2/(N+1))*\mathrm{EWM}A_{\mathrm{yesterday}})$, where *N* = 7 for acute and 28 for chronic. Final results were calculated as follows (Acute EWMA 7/Chronic EWMA 28 = ACWR). Weekly averages of ACWR are presented along with weekly PL totals.

## Results

### Normative values

Table 2 presents the normative values of mean and standard deviation for PL, PL*min^−1^, High-IMA, and Jumps by training period over the year. PL had the highest mean value during the Non-Conference period, followed by Conference, 20-hour preseason, and 8-hour preseason, respectively. PL*min^−1^ did not show much variation between the different periods of the year. High IMA's had the same trend as PL with Non-Conference having the highest values, followed by Conference, 20-hour preseason, and 8-hour preseason, respectively. Jumps were the only variable that had the highest values during the 8-hour preseason, followed by Non-Conference, Conference, and 20-hour preseason, respectively (see Table 2).

**Table 2:** Averages by period.

Period	Player load (mean ± SD)	Player Load/Min (mean ± SD)	High-IMA (mean ± SD)	Jumps (mean ± SD)
8-hour preseason	428.7 ± 169.3	5.4 ± 2.6	19.7 ± 18.7	112.5 ± 106.7
20-hour preseason	468.9 ± 125.0	5.3 ± 1.2	33.8 ± 17.2	76.0 ± 34.7
Non-Conference	532.2 ± 233.9	5.4 ± 1.2	40.3 ± 57.1	95.8 ± 63.3
Conference	496.4 ± 252.5	5.3 ± 1.2	33.8 ± 19.3	80.1 ± 55.3
Yearlong average	492.2 ± 220.8	5.3 ± 1.5	33.7 ± 34.9	88.4 ± 65.6
Period comparisons	Cohen's *d* (95% CI for *d*)	Cohen's *d* (95% CI for *d*)	Cohen's *d* (95% CI for *d*)	Cohen's *d* (95% CI for *d*)
8-hour vs. 20-hour	0.27 (0.08, 0.47)^¶^	0.07 (−0.12, 0.26)	0.79 (0.59, 0.99)^§^	0.48 (0.29, 0.68)^§^
8-hour vs. Non-Conference	0.48 (0.30, 0.66)^§^	0.00 (−0.18, 0.18)	0.43 (0.25, 0.61)^§^	0.21 (0.03, 0.39)^¶^
8-hour vs. Conference	0.29 (0.12, 0.46)^§^	0.08 (−0.09, 0.25)	0.74 (0.56, 0.91)^§^	0.45 (0.28, 0.62)^§^
20-hour vs. Non-Conference	0.32 (0.15, 0.48)^§^	0.11 (−0.05, 0.28)	0.14 (−0.02, 0.30)	0.37 (0.20, 0.53)^§^
20-hour vs. Conference	0.12 (−0.03, 0.28)	0.00 (−0.16, 0.15)	0.00 (−0.16, 0.15)	0.08 (−0.07, 0.24)
Non-Conference vs. Conference	0.15 (0.01, 0.28)^¶^	0.11 (−0.02, 0.25)	0.16 (0.03, 0.30)^¶^	0.27 (0.13, 0.40)^§^

Player Load, Player Load/Min, and High-IMA are in arbitrary units (AUs). Jumps are the raw number of jumps performed. These are the standard metrics for reporting Catapult workload data. Team weekly and/or periods (8-hour preseason, 20-hour preseason, conference, and non-conference) maximum and minimum values can be provided upon request.

IMA, inertial movement analysis; CI, confidence interval.

Numbers in upper panel are presented as mean ± SD; numbers in lower panel represents effect size Cohen's *d* (95% CIs for d); Cohen's d effect sizes of 0.2–0.49 is small, 0.5–0.79 is medium, and 0.8 or higher is large.

^§^ Significant differences between comparisons, where *p *< 0.01.

^¶^ Significant differences between comparisons, where *p* < 0.05.

### Results by training period

Table 2 presents mean and standard deviation values for PL, PL*min^−1^, High-IMA, and Jumps by training period during the year. A linear mixed model analysis revealed a significant PL effect (*p* < 0.01). Further analysis using a Bonferroni post-hoc analysis revealed that PL was significantly lower during the 8-hour period compared to 20-hour (*p *= 0.041), Non-Conference (*p *< 0.01), and Conference (*p *< 0.01) periods, respectively. PL was also significantly lower in the 20-hour period compared to Non-Conference (*p *< 0.01), yet significantly higher in Non-Conference compared to Conference (*p *= 0.03). There were no statistical differences in PL*min^−1^ across the periods of the year, *p* = 0.597. The linear mixed model analysis also revealed a significant period effect on High-IMA (*p *< 0.01). Specifically, High-IMA's were significantly lower during the 8-hour period when compared to 20-hour (*p *< 0.01), Non-Conference (*p *< 0.01), and Conference (*p *< 0.01) periods, respectively, yet significantly higher during Non-Conference when compared to Conference (*p *= 0.032).

Jumps were significantly higher values during the 8-hour period compared to 20-hour (*p *< 0.01), Non-Conference (*p *< 0.01), and Conference (*p *< 0.01) periods, respectively. The 20-hour period had significantly lower Jumps compared to Non-Conference (*p *< 0.01), and significantly more Jumps in Non-Conference compared to Conference (*p *< 0.01).

### Results by weekly loads

**Figure 1:**
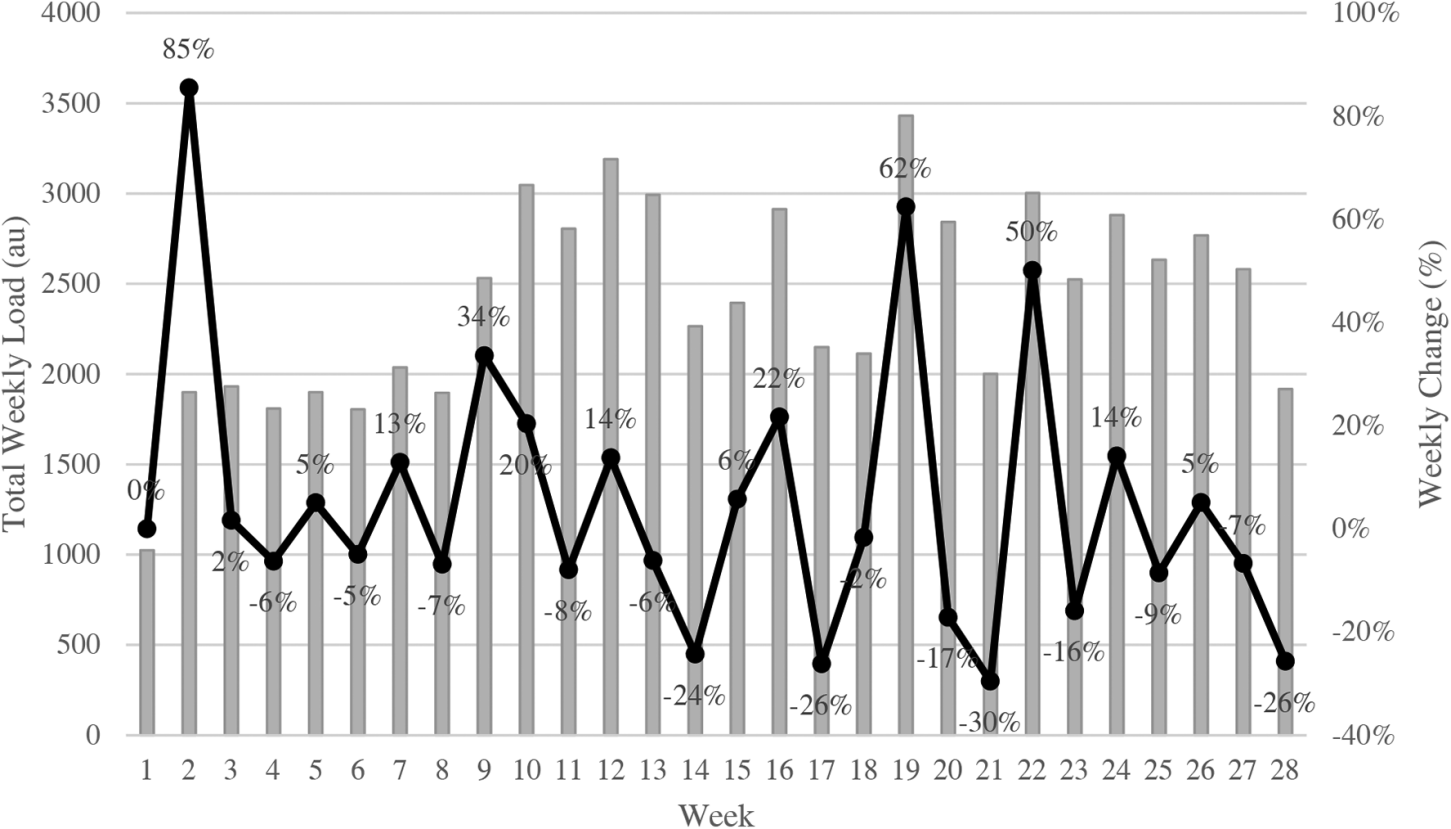
Weekly totals presented in arbitrary units (AU) combined with line graphs that represent percentage difference between weeks in collegiate female basketball players (*n* = 11).

**Figure 2:**
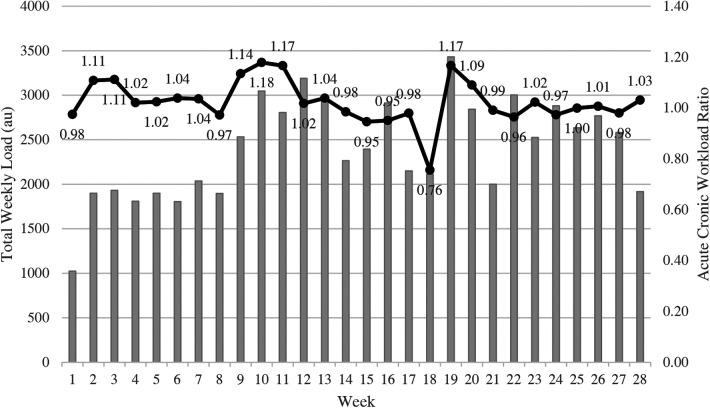
Weekly totals presented in arbitrary units (AU) combined with line graphs that represent acute to chronic workload ratios (ACWR) in collegiate female basketball players (*n* = 11).

Weekly training load totals ranged from a low of 1024 AU during week one, to a high of 3431 AU during week 19 (See Figure 1). Similarly, the highest percent change, week to week, occurred between week one and week two (85%), and the largest percent decrease (−30%) occurred between week 20 and week 21, as shown in Figure 1. Weekly percent change varied every week, however, there were only 5 weeks where a positive or negative percent change, respectively, continued for a second week in a row. ACWR, as shown in Figure 2, varied from a high of 1.18 during week 10 to a low of 0.76 during week 18. Throughout the season, the ratio remained consistent, between 0.95 and 1.04 for 20 of the 28 weeks with only one week < 0.95.

## Discussion

The purpose of this study was to quantify the external workloads of elite collegiate Division I female basketball players over the course of four key periods: 8-hour preseason, 20-hour preseason, Non-Conference, and Conference play. Primary results included (1) establishing norms for PL, PL*min^−1^, High-IMA, and Jumps for each of the 4 collegiate basketball key training periods; (2) determining differences in PL, PL*min^−1^, High-IMA, and Jumps, respectively, for each time-period; and (3) establishing season-long weekly descriptive values for weekly PL totals, weekly percent change in PL, and ACWR.

Establishing normative values for different measures of workload during specific times of the training year is important to plan and apply appropriate training stimuli to facilitate recovery and peaking, and to maximize player availability for training and competition (6, 9, 13). Table 2 shows the average workload values per practice during four key times of the training year.

Whereas comparative data with female basketball players do not exist, Heishman and colleagues studied daily PL data in men's collegiate basketball players over a five-week time block during the 8-hour period (17). PL values averaged 706.5 ± 170.9 AU during the 8-hour period (17), which are considerably higher than the average values found in the present study (428.7 ± 169.3 AU) during the same training period. Male athletes also had higher values for High-IMA, which averaged 62.46 + 20.84, whereas in the present study, PL averaged 33.8 + 19.3. Since practice duration was likely similar between studies based on NCAA regulations, the higher PL and High-IMA found in men (17) is likely attributed to a practice planning variable. For example, previous research indicates that 5v5, 3v3, and full court drills produce higher IMU acceleration load values than 4v4, 2v2, or half court drills, respectively (32). Differences in PL and High-IMA could also be due to differences in study design (i.e., different catapult systems used to collect data, data collection processes and/or data cleaning) or sex differences in leg length, muscle mass, muscular strength, speed, or other performance metrics (30).

Interestingly, PL*min^−1^ was similar between studies. Specifically, PL^.^min^−1^ in Heishman et al. (17) averaged 5.4 ± 1.3 AU in week 1 (the highest value reported), and it averaged 5.4 + 2.6 in the present study across the 8-hour period. This may indicate that when intensity is factored into workload, it is more similar than different between the sexes. Specifically, in the present study, non-activity time (i.e., water breaks and coaching) that exceeded two minutes was excluded from the statistical analysis. In contrast, Heishman et al. (17) started monitoring when players took the floor and ended when practice concluded. This could possibly account for the differences in PL but similar PL*min^−1^ values between studies.

Jumps were also similar between the two studies. Males (17) averaged 103.28 ± 40.48 jumps, and females in the present study averaged 112.5 + 106.7 jumps. There appears to be more individual variability in jumping in the present study compared to the study by Heishman and colleagues (17), based on the differences in standard deviations recorded. In addition, jumps are measured without consideration of jump height.

While some of the measured variables differed between collegiate athletes in the present study and that of Heishman et al. (17), previous research examining workloads in male and female elite junior basketball players during competition (27) (43 ± 27 AU in males, 39 ± 21 AU in females, *p* = 0.69) concluded that differences were not statistically different, and effect sizes were small (28). Additional research should aim to determine if workload variables consistently vary between males and females—when using standardized data collection and analysis procedures–or whether differences increase as athletes mature or move to more advanced levels of play.

While averages for each variable during each time period in the present study can provide valuable context for programming, standard deviations provide insight into the variations throughout the seasonal periods and between athletes. Variations in PL between athletes, even when participating in the same drills, is likely due to physical and playing style differences, as well as differences in leg length and strength (30, 33). Therefore, standard deviations can also provide insight into individual PL ranges that may be seen in collegiate women's basketball. While standard deviations should not be examined as a key programing variable, they can provide valuable insights into differences between players in training intensity during similar time periods.

Our second study objective was to describe the workload requirements of each training period: 8-hour, 20-hour, Non-Conference, and Conference. To our knowledge, no other study has compared PL, PL*min^−1^, High-IMA, and Jumps across time periods over the course of a year; however, one study examined these variables during game-play over the course of four years (29). In the present study, PL, High-IMA, and Jumps were all significantly different between time periods *p* < 0.05. PL*min^−1^ was the only variable that did not show a significant difference across any of the periods. It is possible that the average intensity of the practices stayed constant while duration–and therefore PL–varied. PL and High-IMA's were significantly lower in the 8-hour period compared to every other time period (*p* < 0.05) due to the substantially reduced time allowed for practice during the 8-hour period. Interestingly, Jumps were significantly higher in the 8-hour period compared to the other periods (*p *< 0.01 for all). This is likely due to the incorporation of planned plyometric sessions during the 8-hour period that were not performed during other times of the year. Specifically, the jump loads were planned as low-level plyometrics as part of the warm-up during the 8-hour period of training. These jump loads were incorporated into practice to increase the volume of game-like jumps and landings during the 8-hour period where time was limited. In addition, training data suggested the largest percentage change in training volume (85%) was between weeks 1 and 2–when Jumps were also the highest.

PL was significantly higher during the Non-Conference period compared to the 8-hour (*p *< 0.01), 20-hour (*p *< 0.01), and Conference (*p* < 0.05) periods, respectively. Game play explains these differences to a certain extent, yet it does not explain the difference in PL between Conference and Non-Conference periods. While Conference and Non-Conference periods include practices and games, game days and travel vary between the two periods. During Conference play, all games are played on Fridays and Sundays, whereas Non-Conference games can be played any day of the week. This could lead to increased practice volume when there is a shorter break between a cluster of games. Also, PL during the Conference period could be lower due to a planned taper in order to keep players fresh and manage injuries as the team prepares for post-season play.

The third objective in the present study was to describe weekly values for workload totals, percent change in workload, and ACWR throughout the season. While large-scale averages and comparisons between time periods are important, examining loads over a shorter time period (e.g., week to week) can also improve the planning process. Previous research has found that percent change as well as ACWR are valuable tools coaches can use to examine the change in training load across weeks (13, 33, 36). While injury data were not examined in the present study, previous research indicates that a weekly increase in PL above 15% can lead to a 28%–49% increase in injury risk (13). Increases in weekly PL above 15% occurred in the present study in 13 of the 28 recorded weeks and the largest difference occurred between week one and week two; however it should be noted there was one less training session in week one by design. Our values are considerably higher than in another study that examined workloads in professional Lithuanian women's basketball players throughout a season; in their study only 3 of the 24 weeks had a greater than 15% change in workload (25). In contrast, a study examining workloads in collegiate male basketball players reported 5 of the 10 weeks had an increase in PL greater than 15% (9). This could indicate that inconsistencies in scheduling within basketball programs make it harder to keep PL consistent week to week. Collegiate basketball is also restricted by NCAA regulations, which could affect how workloads are increased throughout the year.

ACWR can also provide coaches with information about athlete training load variations. In the present study, there was a consistent weekly ACWR range between 0.95 and 1.17 for all but one occasion, where there was a low of 0.76. This deviation could be explained by a holiday break where athletes did not participate in any mandatory activities with the team, and these results are similar to those from a study examining professional Lithuanian women's basketball players near the end of December (25). While injury risk is always present, research has found that the optimal range for decreasing this risk is 1–1.5 ACWR (19, 33, 36). In the present study, 17 of the 28 weeks were within the recommended ACWR range, with the other weeks having only minor deviations. This was similar to previous measures of ACWR in elite women basketball players where ACWR values ranged from 0.9 and 1.1 for most weeks (25).

The findings from the present study can be applied to day to day, week to week, and long-term practice planning to better maximize practice time and minimize risk of injury in collegiate women's basketball players. By utilizing averages and ranges of IMU-based workloads, coaches can create an outline of the collegiate basketball training season and adapt it to the unique needs of a team, paying particular attention to transitions between specific time periods and week-to-week changes in workloads.

To our knowledge, this study was the first to report season-long descriptive IMU-based workload data in collegiate women's basketball players from a Power 5 conference. Nevertheless, there are limitations to consider. First, a relatively small sample size of 11 athletes from a single team was utilized. As such, results are specific to this team and may vary based on coaching style, schedule, fitness, age, and data analysis. Second, results may also vary based on player position (i.e, guards tend to have higher workload values compared to posts) (29), or on an individual's gait, biomechanics, or speed of locomotion (15). Thirdly, there are a variety of critiques (3, 14, 20, 21, 37) related to the use and measurement of ACWR as a predictor of injury risk (e.g., ACWR is fundamentally flawed, should not be used to predict injury risk, and should be dismissed or re-evaluated). Most agree that AWCR is best utilized as a part of a multifactorial approach to examine athlete well-being, performance, and fatigue (3, 37), and that predicting risk of injury warrants additional research to establish causality (14, 20, 21). Fourth, these data do not include workouts in the weight room or conditioning that occurred outside of practice time, and we did not control for workouts outside of organized team practices. Therefore, caution should be used when comparing results from the present study to other teams or levels of women's basketball.

Future research is needed to continue to profile athletes and provide longitudinal data in women, notably in elite women's basketball. A recent systematic review (3) tracked 2375 injuries in 1,234 male athletes, who participated in Rugby, Australian Football, Soccer, and Cricket. There are very few studies that examine IMUs in female athletes, and no systematic reviews that link workload to athlete fatigue, performance, or injury risk in women because to date, research is mostly limited to men in elite and professional sports. Future research with both men and women should work to develop standardized multifactorial methods to examine the association between variables such as workload, sleep, stress, travel, hydration, and nutrition—and basketball statistics, performance, and non-contact injuries (3, 37). Accounting for moderators such as previous injuries, fitness, fatigue, and mood is also important (3, 37), and points to the need to individualize player profiles when examining data. When examining injuries, the impact of tissue injured, type of injury and grading of injury (i.e., seriousness) on ACWR, should also be considered (3).

While developing standardized multifactorial methods, researchers should continue to test recommended athlete workload monitoring protocols for various sports, levels of participation, fitness and performance metrics, and sex-differences; or re-evaluate the ACWR models–because clear consensus has not been reached on the best approach (38). For example, although a 1 : 4 week split is currently the most common timeframe used to calculate ACWR, other strategies could be examined and recommended based on game schedule, time in season, and training. The impact of latent workload is important and should continue to be tested using EWMA adjusted ACWR (38).

A third area for future research is to expand the definition of neuromuscular fatigue from jumps alone to variables that include Flight Time to Contraction Time Ratio (FT:CT) and Reactive Strength Index, Modified (RSI_mod_), as specified in Heishman et al. (17). These data would provide coaches and trainers with additional jump-related fatigue metrics for consideration. In addition to examining the aforementioned jump-related fatigue metrics, viewing fatigue as a multifactorial concept that includes travel, sleep, hydration, and academic or other stress would help further define the relationship between fatigue and performance (27). In the present study, loads were examined based on the four periods of the training season, however, load changes experienced in practice on a day-to-day basis require further examination.

Two additional areas for future research include continuing to study inter-device reliability and various types of validity (18, 24), and expanding workload variables to include distance and speed, and re-examining fitness metrics regularly (as they change throughout the training cycle or year) to determine the impact of improved fitness on factors that determine athletic success (3).

## Practical applications

The present study helps sport and performance coaches to understand the physical demands of players across a collegiate women's basketball season. By understanding how training loads can be measured and quantified, and by using seasonal and period-specific reference values as part of a multifactorial athlete profile, coaches can objectively plan and track training demands and vary loads based on time of year and short-term and long-term goals. The normative values in the present study quantify average player load, variations between players, and provide a comparison of the loads during the four key periods of the year. This allows coaches to understand the changes in demands of an upcoming transition period and plan accordingly. By utilizing metrics such as percent change and Acute to Chronic Workload Ratios, along with other data (e.g., sleep, stress, level of fitness, etc.), coaches can create a plan based on reference ranges while also allowing for team-specific adjustments. Use of multifactorial data facilitates educated decision-making that can impact a team's programming throughout the year.

While data in the present study are valuable to understand, coaches should recognize that other variables will vary, and every team (and even individual athletes) will present with their own unique team and player profiles that may differ from those found in this study.

## Conclusion

Data on female athletes is sparse, and there is a need to better quantify training and performance loads at various points throughout the season. Our study quantified and profiled the external load of practice and games during a Power-5 DI Women's Collegiate Basketball season. ACRW varied throughout the season, with most of the changes within the range recommended to minimize injury risk. These findings, combined with other research on workloads during women's basketball, can be used to describe the physical demands across a season, and coaches should be able to use these data as part of a multifactorial approach to more effectively plan practices and game strategies.

## Data Availability

The raw data supporting the conclusions of this article will be made available by the authors, without undue reservation.
